# Symptomatic Patient With Two Separate Accessory Navicular Bones

**DOI:** 10.7759/cureus.47881

**Published:** 2023-10-28

**Authors:** Brandon Sharkey, David Yatsonsky, Nickelas Huffman, Tony Dong, Adrian Lewis

**Affiliations:** 1 Department of Orthopaedic Surgery, University of Toledo College of Medicine and Life Science, Toledo, USA; 2 Department of Pediatric Orthopaedic Surgery, ProMedica Toledo Hospital, Toledo, USA

**Keywords:** accessory navicular bone, geist classification, surgical excision, os naviculare treatment, bipartite os naviculare

## Abstract

We present the case of a symptomatic patient with two separate accessory navicular bones, a novel finding that does not fall within current classification standards. Furthermore, there is a paucity of current literature with regard to the management of symptomatic cases. Accessory navicular bones, sometimes referred to as os naviculare, are ossicles that can occur in multiple configurations and are considered developmental anomalies. The accessory navicular is an accessory bone found on the medial side of the navicular bone of the foot. While often asymptomatic, they can occasionally lead to clinically significant pain and/or deformity that can cause patients to seek out treatment and sometimes surgical correction. Diagnosis relies on clinical suspicion and imaging studies.

A nine-year-old female patient presented initially with complaints of sharp pain over the medial side of her left foot, after which X-rays were obtained that demonstrated an accessory navicular bone. Upon diagnosis, conservative measures were implemented, including placing the patient in a short-leg cast with limited activity. After a failed trial of conservative measures, the patient underwent surgical excision of the accessory navicular bone, with imaging and intraoperative findings of two separate accessory navicular bones, a unique finding in patients with accessory navicular bones. During the six-week follow-up, the patient improved with no new complaints or concerns and was informed she could begin weight-bearing as tolerated with two crutches; she was then weaned from the crutches and returned to normal activity. In the current case, we followed the same route of treatment used to treat a single accessory navicular bone, utilizing first non-surgical interventions and then ultimately surgical excision after continual pain despite conservative measures. This case highlights the promising outcome for a patient with two separate accessory navicular bones when following the guidelines for the treatment of a single accessory navicular bone.

## Introduction

Accessory navicular bones are a relatively common pathology with higher prevalence in females and are often painful and tender in teenagers and patients in their twenties [1,2]. Reports have shown that 4%-21% of the population has an accessory navicular bone [1,3-5]. Many patients with an accessory navicular bone have a tibialis posterior tendon that has subluxated over the medial malleolus, and the tendon has to pull harder to maintain a satisfactory effect, which frequently induces a stress fracture [2]. The arch of the foot becomes lower as the patient sustains a traumatic division of the tibialis posterior tendon [2,6].

There is still little consensus on why some patients with os naviculare are symptomatic while others remain asymptomatic [7]. While some authors speculate symptoms arise from stress fractures related to a subluxated tibialis posterior tendon, others have identified associations with the pes planus and an associated increase in stress on the accessory navicular during weight-bearing activities [5,8].

The accessory navicular bone is classified using Geist classification [9] based on three different subtypes established from the work of Emil S. Geist. Classifications were later updated and refined by Lawson et al. [2] and Sella et al. [10]. The accessory navicular bones can be classified as type I, II, or III. A type I accessory navicular is characterized by a 2-3 mm sesamoid bone embedded within the tibialis posterior tendon that is anatomically separate from the navicular and is generally asymptomatic [6]. The larger type II accessory navicular is a triangular or heart-shaped ossification center measuring up to 12 mm, is found medial to the navicular tuberosity, and may be associated with a painful foot in certain populations, such as athletic adolescents [2]. The type III accessory navicular is identified by a solid navicular tuberosity, and it may represent a fused type II accessory bone. Type III navicular os are generally asymptomatic, similar to type I os unless associated with bunion formation [2,6].

Conservative treatment often involves arch support, which lifts the arch to reduce pressure placed on the os from the shoe, resulting in symptomatic improvement in some cases [2,3,11,12]. When pain and symptoms persist, usually for one to two years, there is merit in surgical excision [11,13]. Only a small percentage of patients will need surgical intervention, but surgical outcomes are typically very successful with the excision of the accessory navicular bone. An article by Macnicol and Voutsinas reviewed the results of excision in Edinburg, where a population of 21 underwent excision of the os naviculare and the main navicular bone was contoured to prevent any residual prominence. Twenty-six other patients had a Kidner procedure with similar results, and overall, 86% achieved patient satisfaction [14].

While the Geist classification of accessory navicular bones [9] has been overall effective at defining most accessory navicular subtypes, this case report presents a previously undefined presentation of an os naviculare with two distinct ossification centers.

## Case presentation

**Figure 1:**
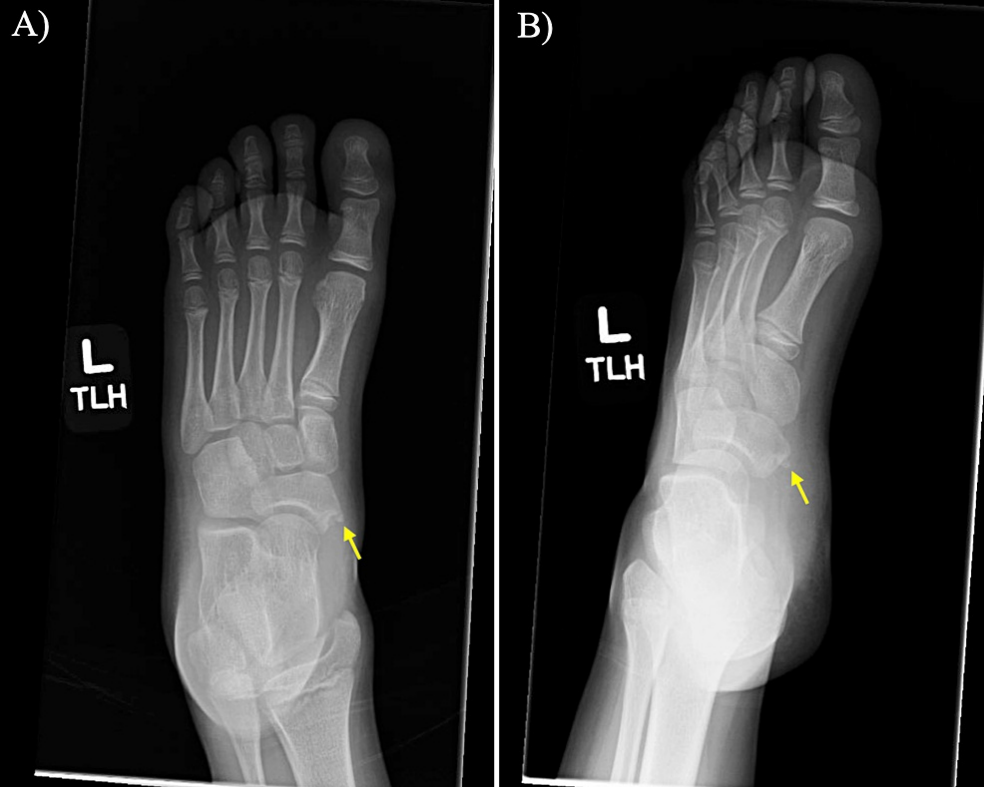
Preoperative foot and ankle radiographs A) Anterior-posterior (AP) view X-ray of the left foot demonstrating avulsed ossification center (yellow arrow) and possible second ossification center at the time of initial presentation; B) Oblique view X-ray of the left foot at the time of initial injury demonstrating fractured navicular ossification center (yellow arrow).

A nine-year-old female with a past surgical history of right index pollicization and dermal sinus resection presented to our clinic with an injury to the left ankle and foot after landing awkwardly while jumping on a trampoline. One day after the injury, the patient was seen at an urgent care facility, where she received X-rays and a recommendation to follow up at our clinic. The patient presented to our clinic several days after the initial injury. During the patient’s first visit to our clinic, she complained of a sharp pain over the medial side of the left foot. The X-rays obtained at the urgent care facility demonstrated an accessory navicular bone with a likely avulsion fracture (Figure 1).

Conservative measures were the initial plan of action, which involved placing the patient in a short-leg cast, with the patient remaining weight-bearing with limited activity. The patient was scheduled for follow-up in four weeks. However, before her follow-up appointment, the patient notified our office that she was experiencing pain while walking in the cast. It was recommended that she avoid walking if it causes pain, with the plan remaining as a follow-up in four weeks from the initial cast placement.

The patient returned to the office for a four-week follow-up and X-rays of the left foot. The X-rays displayed interval healing at the navicular bone in the accessory area. No further displacement was noted, and the cast was removed. The patient had no pain, was not taking any pain medication, and was tolerating normal daily activities. Therefore, the patient no longer required immobilization but was counseled on the importance of limiting intense physical activity for several weeks. She was instructed to follow up with our office on an as-needed basis.

**Figure 2:**
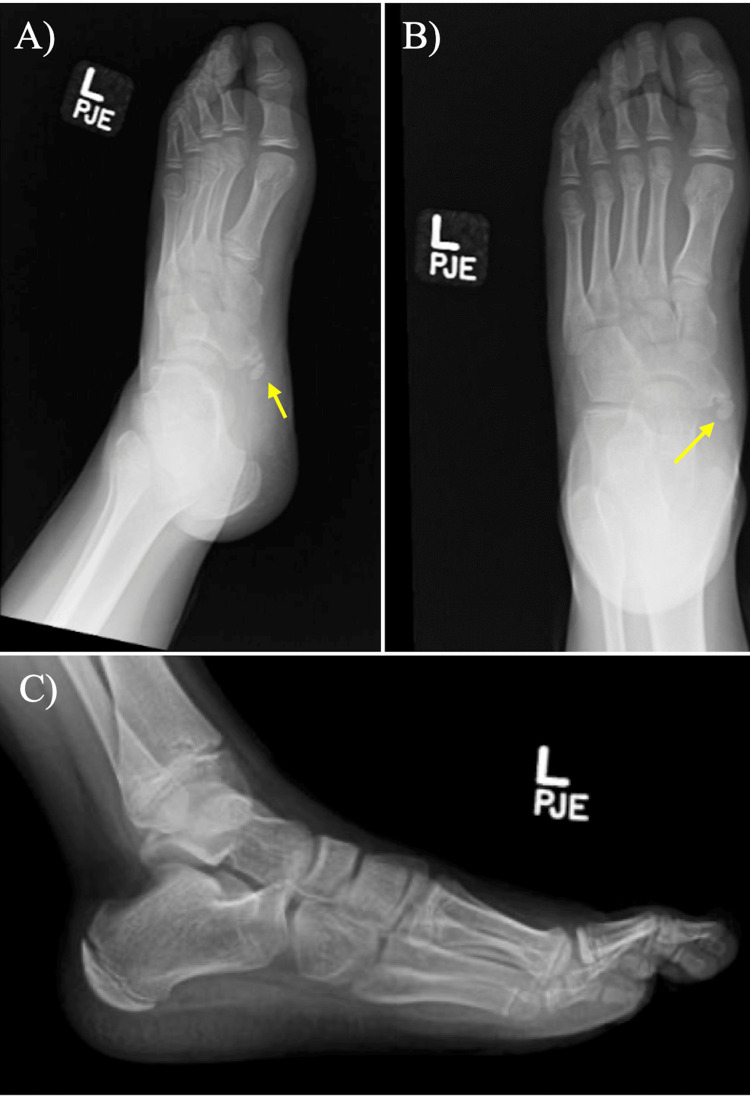
Repeat preoperative foot and ankle radiographs. A) Oblique view X-ray of the left foot, six months post-injury, demonstrating two distinct ossification centers (yellow arrow); B) Anterior-posterior (AP) view of the left foot, six months post-injury, demonstrating two distinct ossification centers (yellow arrow); C) Lateral view X-ray of the left foot. Ossicles are occluded by the remainder of the foot.

Ultimately, recurrent pain and discomfort developed shortly after cast removal. The pain continued to increase in intensity and peaked roughly eight weeks after the removal of the cast; the patient was therefore placed in a boot, but this offered no improvement in symptoms. Due to the continual pain, the patient underwent repeat radiographs, which showed continued demonstration of ossicles, more pronounced than at the time of injury (Figure 2).

To the best of the authors’ knowledge, there is currently no literature providing an explanation for the increased visibility of accessory navicular bones over time. Furthermore, the authors do not have an explanation for this interesting finding. However, it is possible that the accessory navicular was more pronounced in follow-up X-rays due to variations in radiologist technicians and the angles at which the X-rays were obtained.

The patient continued to describe unrelenting pain across the medial aspect of the foot, near the area where she was known to have an accessory navicular. She was able to bear weight, although this caused her intense pain. The use of over-the-counter medications and ice offered minimal improvement. Given the lack of improvement with conservative treatment, the patient wished to proceed with surgical intervention. Surgical consent was obtained, and surgery was scheduled. Imaging indicated that there were two separate ossification centers, which did not fit into a given classification system. This did not alter our plan to pursue surgical management, and the decision was made to proceed.

**Figure 3:**
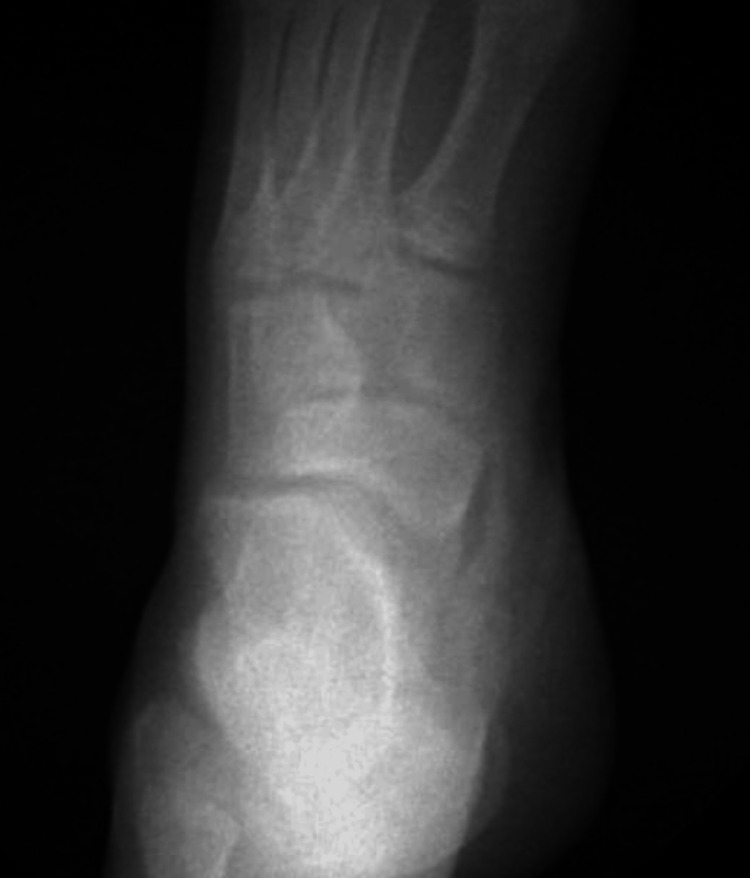
Intraoperative foot and ankle radiograph Intraoperative anterior-posterior (AP) view X-ray of the left foot following excision of the ossicles, demonstrating complete excision of the ossicles and no new osseous abnormalities.

During surgical excision of the accessory os naviculare, it was observed that the patient had two separate accessory navicular bones consistent with type I and type II classifications according to the Geist classification system [9]. These were excised uneventfully and confirmed on intraoperative fluoroscopy (Figure 3).

The tibialis posterior tendon was repaired to the dorsal cuff, wounds were closed with polydioxanone (PDS) and monocryl, and the patient was cast with the foot in inversion and followed up as an outpatient.

Six weeks after the surgery, the patient was asymptomatic with no complaints or concerns. The cast was removed, and the patient was informed that she could weight-bear as tolerated with two crutches, then one crutch, then finally advancing to weight-bearing with no crutches. The patient was informed of the importance of participating in physical therapy. In addition, the patient was instructed to refrain from high-impact activities until strength and range of motion had been regained.

## Discussion

The current case presents a novel finding of two separate accessory navicular bones. However, despite these unique findings, it is important to note this patient’s promising improvement utilizing the algorithm commonly followed to treat a single accessory navicular bone. Specifically, we first utilized conservative treatment and then proceeded to surgical excision after the patient experienced persistence of symptoms. Therefore, although our patient had the unique finding of two accessory navicular bones, optimal treatment may be similar to that used for a single accessory navicular bone.

Often, os naviculare is clinically insignificant, with a majority of cases remaining asymptomatic [15]. However, the presence of os naviculare can cause pain, often in pediatric and young adult populations, due to the tension and compressive forces transmitted across the posterior tibial tendon [15]. Similarly, as in our case, it is important to note that type II is the most common type to cause midfoot pain [16].

Our patient was unique in that she had bipartite ossification centers, resulting in her os navicular classification as both type I and type II. However, despite the uniqueness of this case presentation, having bipartite ossification centers changes neither the management plan nor the outcome for patients. This offers an important contribution to the literature because it offers healthcare providers a case presentation of a patient who achieved excellent postoperative outcomes following the same treatment algorithm used when treating patients with a single accessory navicular bone. In other words, according to the postoperative outcomes in our case, healthcare providers should not alter treatment strategies if they discover a patient with two separate accessory navicular bones.

Treatment of symptomatic accessory navicular is first non-operative, using more conservative measures such as non-steroidal anti-inflammatory drugs (NSAIDs), immobilization, and physical therapy. When conservative measures fail, surgical intervention may be necessary. However, the number of patients who require surgical intervention is minimal. Although roughly one in five of the population may have an accessory navicular bone [1,3-5], only one in 1,000 adult patients develop symptoms from the presence of the os naviculare [7]. Of the patients that develop symptoms, one study found that only 30% of symptomatic patients require surgical intervention [17]. When surgical intervention is required, surgery has been shown to have excellent outcomes, with one study showing 14 of 14 patients having improvement in pain after undergoing excision of accessory os naviculare [12]. In addition, this study showed an improvement in the American Orthopaedic Foot and Ankle Society (AOFAS) Midfoot Score [18] from 48.2 preoperatively to 94.5 postoperatively [12].

We followed this same route of treatment, utilizing the first non-surgical interventions for our patient’s bipartite ossification center. After continual pain despite conservative measures, surgical excision of the ossicles was indicated. Following surgery, our patient was non-weight-bearing for six weeks, and overall, the surgery and postoperative plan resulted in drastic improvement in our patient’s pain.

## Conclusions

Bipartite os naviculare is a rare but possible occurrence in pediatric and adult populations. We emphasize the importance of a thorough review of imaging prior to surgery, along with a thorough dissection intraoperatively, in order to better ensure the removal of all symptomatic accessory tissue causing pain to the patient. We strongly recommend against computed tomography (CT) and magnetic resonance imaging (MRI) preoperatively in this case to reduce radiation and cost, but care needs to be taken to avoid missing possible secondary ossification centers in ossicle excision surgeries of accessory navicular bones. If a patient is found to have two separate accessory navicular bones, healthcare providers can utilize the same treatment strategy that is utilized for a single accessory navicular bone: first, utilization of conservative treatment options such as activity limitations, arch support, and over-the-counter pain management; then, if symptoms persist, discussion with the patient of possible surgical excision of the symptomatic accessory navicular bone.
